# Design, Analysis, and Implementation of a Novel Biochemical Pathway for Ethylene Glycol Production in *Clostridium autoethanogenum*

**DOI:** 10.1021/acssynbio.1c00624

**Published:** 2022-05-11

**Authors:** Barbara Bourgade, Christopher M. Humphreys, James Millard, Nigel P. Minton, M. Ahsanul Islam

**Affiliations:** † Department of Chemical Engineering, Loughborough University, Loughborough LE11 3TU, U.K.; ‡ BBSRC/EPSRC Synthetic Biology Research Centre, Biodiscovery Institute, University of Nottingham, Nottingham NG7 2RD, U.K.

**Keywords:** ethylene glycol, synthetic pathway, metabolic engineering, synthetic biology, Clostridium autoethanogenum

## Abstract

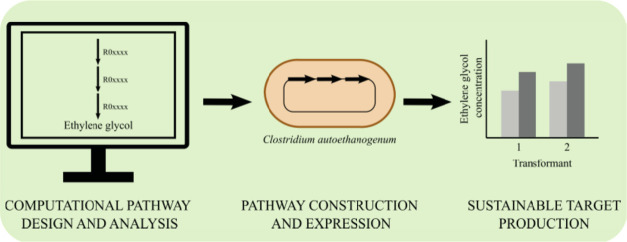

The platform chemical ethylene glycol (EG) is used to manufacture various commodity chemicals of industrial importance, but largely remains synthesized from fossil fuels. Although several novel metabolic pathways have been reported for its bioproduction in model organisms, none has been reported for gas-fermenting, non-model acetogenic chassis organisms. Here, we describe a novel, synthetic biochemical pathway to convert acetate into EG in the industrially important gas-fermenting acetogen,*Clostridium autoethanogenum*. We not only developed a computational workflow to design and analyze hundreds of novel biochemical pathways for EG production but also demonstrated a successful pathway construction in the chosen host. The EG production was achieved using a two-plasmid system to bypass unfeasible expression levels and potential toxic enzymatic interactions. Although only a yield of 0.029 g EG/g fructose was achieved and therefore requiring further strain engineering efforts to optimize the designed strain, this work demonstrates an important proof-of-concept approach to computationally design and experimentally implement fully synthetic metabolic pathways in a metabolically highly specific, non-model host organism.

## Introduction

Synthetic biology, through the application of genetic and metabolic engineering technologies, contributes to the development of sustainable bioprocesses, enabling the bioproduction of value-added commodity chemicals from renewable resources. This discipline is essential to reduce our dependence on fossil fuel-based petrochemical industries, which negatively impact the environment and significantly contribute to the current climate emergency through greenhouse gas emissions.

As more bioprocesses are being developed, many industrially important target chemicals can now be produced with microorganisms. For example, the platform chemical ethylene glycol (EG) is an important industrial solvent that is widely used as an antifreeze agent and a precursor for several polyesters such as the plastic, polyethylene terephthalate.^1−3^ Considering its high market value and demand with an estimated global production of 65 million tons in 2024,^4^ sustainable EG bioproduction is highly beneficial as compared to traditional fossil fuel-based chemical processes from both industrial and environmental points of view. In fact, several EG-producing biochemical pathways have previously been implemented in model organisms. EG production, for instance, has been reported from xylose in *Escherichia coli*(5−7) and *Saccharomyces cerevisiae*,^8−10^ from serine in *E. coli,*(11) and from glucose in *Corynebacterium glutamicum*.^12^ The challenges and progresses made toward EG bioproduction have been reviewed elsewhere.^13^ More recently, *Enterobacter cloacae* has been identified as a natural EG producer,^14^ with xylose as the main substrate. These studies highlight the research efforts invested toward sustainable EG bioproduction, crucial for the industry.

While the synthetic biology and metabolic engineering progresses described above are imperative to move toward a sustainable future, non-model microorganisms with specific substrate utilization capabilities are also particularly promising for industrial applications. Indeed, it can be argued that other substrates, such as C1-gases (CO_2_ and CO) and synthesis gas (a mixture of CO_2_, CO, and H_2_), are superior to sugars, as they are widely available from a diverse range of waste feedstocks,^15^ and their availability does not compete with limited arable land or crop production. For example, acetogens, the Gram-positive anaerobic bacteria, show great potential for industrial applications due to their ability to grow autotrophically via the Wood–Ljungdahl (WL) pathway.^16,17^ This metabolic property allows them to use CO_2_ or CO as their sole carbon source to synthesize acetyl-CoA, further converted into acetate and other species-specific products such as ethanol via gas fermentation. Considering their diverse metabolic abilities and potential for industrial applications, research efforts in the past decade have been focused on developing genetic tools for acetogens^18−21^ and applying them for metabolic engineering purposes,^22−24^ allowing sustainable production of several value-added chemicals while fixing CO_2_. The mesophilic acetogen, *Clostridium autoethanogenum,*(25) has previously been modified with CRISPR-Cas approaches^26^ and other genetic tools^27−29^ for improved ethanol production^30^ or the production of non-native targets.^31^ Due to its attractive metabolic properties, *C. autoethanogenum* stands out as a key chassis organism for industrial bioprocesses.

To further expand the repertoire of products that can be synthesized by microorganisms, new-to-nature or synthetic metabolic pathways can be designed with various computational tools.^32^ In fact, many *de novo* pathways have been reported in the literature for a range of target products and chassis organisms.^33−35^ For example, novel pathways for EG production from acetyl-CoA were previously designed for the two acetogens, *Moorella thermoacetica* and *Clostridium ljungdahlii*.^36^ Although, in theory, computationally designed synthetic pathways could allow the bioproduction of virtually any target chemical, experimental implementation remains challenging due to suboptimal enzyme kinetics and difficult gene expression in host organisms, as discussed elsewhere.^37^ As such, fewer studies report successful synthetic pathway implementation following computational design and analysis, further highlighting the remaining gap between computational approaches and experimental applications.

This study describes the detailed computational design, analysis, and experimental implementation of a novel, synthetic biochemical pathway for EG bioproduction from acetate in *C. autoethanogenum*. The results discussed here clearly show that computationally designed biosynthetic pathways, selected with rational pruning criteria and further analyzed for pathway feasibility and host compatibility, are functional in a chosen host organism. Although the product yields reported in this study remain insufficient for direct industrial applications of the designed chassis and would require further strain engineering and pathway optimization efforts, this proof-of-concept study is very encouraging for the metabolic engineering of gas-fermenting acetogens and opens the door for other high-value target chemicals manufacture by using these industrially important, attractive microbial chassis.

## Materials and Methods

### Pathway Design and Analysis

Synthetic metabolic pathways from acetate to EG were designed with the cheminformatics tools, From Metabolite to Metabolite (FRM)^38^ and Metabolic Route Explorer (MRE).^39^ A preselection process was applied based on the following pathway-pruning criteria to choose the best candidate pathway: pathway length; requirement for external metabolites; gene availability; and gene origin, further discussed in the Results and Discussion section. The chosen candidate pathway was then further analyzed with *C. autoethanogenum* genome-scale metabolic model^40^ and the Flux Balance Analysis (FBA) tool^41^ in the COBRA toolbox.^42^ The group contribution method (GCM)^43^ and the online tool, eQuilibrator^44^ were explored for a thermodynamics-based feasibility analysis of the designed synthetic pathway for EG manufacture.

### Bacterial Strains and Growth Conditions

The bacterial strains and plasmids used in this study are listed in Table 1. *E. coli* TOP10 and sExpress^45^ were grown in Luria–Bertani (LB) medium at 37 °C. LB was supplemented with 15 g/L of agar for plates. The medium was also supplemented with 500 μg/mL of erythromycin, 25 μg/mL of chloramphenicol, and 50 μg/mL of kanamycin where appropriate. *C. autoethanogenum* DSM 10061 C24 (henceforth *C. autoethanogenum* C24)^46^ and its derivatives were grown in YTF (10 g/L yeast extract, 16 g/L tryptone, 10 g/L fructose, 0.2 g/L sodium chloride, 1 mL vitamin solution, 1 mL trace element solution, pH 5.8), solidified with 15 g/L of agar and supplemented with 6 μg/mL of clarithromycin and 7.5 μg/mL of thiamphenicol when needed. 5 mM of β-lactose and 5 mM of theophylline were added to induce gene expression when required. *C. autoethanogenum* C24 and its derivatives were grown at 37 °C in a Don Whitley anaerobic chamber (Don Whitley Scientific, UK).

**Table 1:** Bacterial Strains and Plasmids Used in This Study

name	description	reference
Bacterial strains		
*E. coli* TOP10	cloning strain	Invitrogen
*E. coli* sExpress	conjugation donor strain	(45)
*C. autoethanogenum* C24	*C. autoethanogenum* DSM 10061 with genome-integrated lactose-inducible tcdR regulator	(46)
*C. autoethanogenum* C24 EG	*C. autoethanogenum* C24 carrying pMTL83251-EG and pMTL84151-fucO	this study
Plasmids		
pMTL83251	shuttle vector; pCB102 replicon; *ermB*	(56)
pMTL84151	shuttle vector; pCD6 replicon; *catP*	(56)
pMTL83251-EG	pMTL83251 with the synthetic operon *aceA*-*ghrA*-*aldA* controlled by the P_*tdcB*_ promoter	this study
pMTL84151-fucO	pMTL84151 with *fucO* controlled by the theophylline-inducible riboswitch^47^	this study

### Plasmid and Strain Construction

The four genes: *aceA*, *ghrA*, *aldA*, and *fucO* were amplified from *E. coli* genomic DNA with Q5 High-Fidelity DNA polymerase (New England Biolabs, UK). The primers, synthesized by Sigma-Aldrich, are listed in Table S1 in the Supporting Information. *aceA*, *ghrA*, and *aldA* were cloned into pMTL83251 with SacI-SpeI, SpeI-HpaI, and HpaI-XbaI, respectively, downstream of the P_*tdcB*_ promoter.^46^*fucO* was fused to the riboswitch-P_*fdx*_ promoter^47^ in an NEBuilder HiFi DNA Assembly (New England Biolabs, UK) reaction and cloned into pMTL84151 with NotI and NheI. Cloning steps were performed in *E. coli* TOP10. The plasmids were then transformed into *E. coli* sExpress for conjugation into *C. autoethanogenum* C24 as previously described.^45^ pMTL83251-EG was first introduced into *C. autoethanogenum* C24, and pMTL84151-*fucO* was conjugated in a second conjugation step. Plasmids and transformants were confirmed by Sanger sequencing by Eurofins Genomics (Eurofins Genomics Germany GmbH). *C. autoethanogenum* C24 strains were stored in cryotubes at −80 °C in 15% dimethyl sulfoxide.

### Cultivation and Product Analysis

*C. autoethanogenum* C24 EG was grown in YTF, supplemented with 6 μg/mL of clarithromycin and 7.5 μg/mL of thiamphenicol at 37 °C in an anaerobic chamber (Don Whitley Scientific, UK). 5 mM of β-lactose and/or 5 mM of theophylline was added to the cultures when required for induction of promoters. At each timepoint, OD_600_ was measured for growth curves, and 1 mL of culture was collected and centrifuged at 13,000*g* for 5 min. Supernatants were stored at −20 °C in cryotubes until HPLC analysis was performed. Supernatant samples were diluted 1:1 with 50 mM valerate in 0.005 M sulfuric acid; after vortexing, each sample was filtered into a HPLC vial. The analysis of metabolites was performed using a Thermo Scientific Ultimate 3000 HPLC system equipped with UV/vis and RI detectors and an Aminex column (300 × 7.8 mm, 9 μm particle size) (Bio-Rad laboratories) kept at 35 °C. Slightly acidified water was used (0.005 M H_2_SO_4_) as the mobile phase with a flow rate of 0.5 mL/min.

## Results and Discussion

### Computational Design and Analysis of Synthetic Pathways

**Figure 1:**
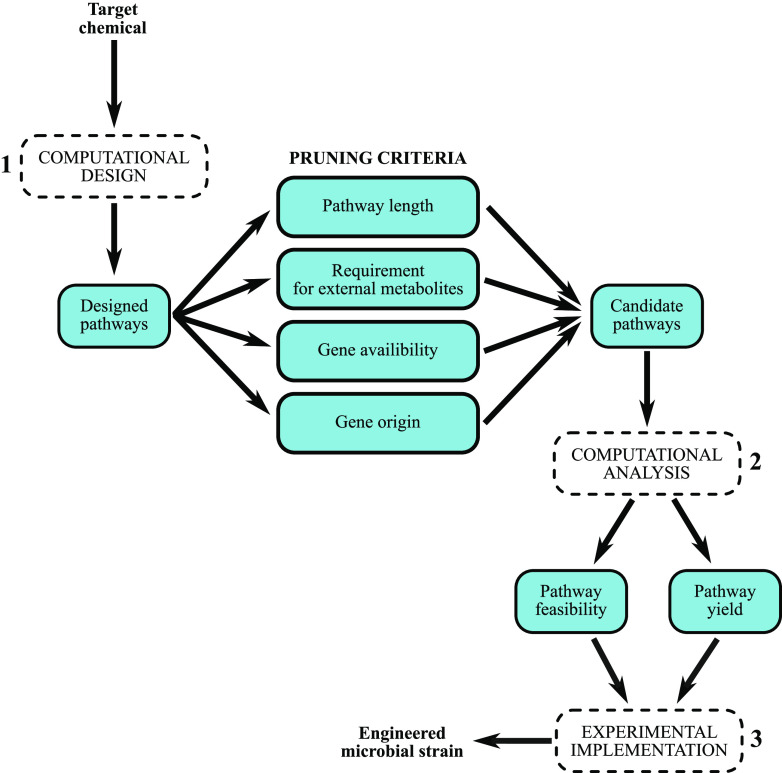
Workflow developed to engineer*C. autoethanogenum* to produce EG with a novel, synthetic metabolic pathway. (1) First, computational cheminformatics tools were used to design novel biosynthetic pathways, which were further curated using several pruning criteria. (2) The candidate pathways were then analyzed with*C. autoethanogenum*GEM to predict pathway feasibility and yield. (3) Finally, one candidate pathway was experimentally implemented and constructed in*C. autoethanogenum*, leading to EG production by this organism.

Synthetic biology, mediated through genetic and metabolic engineering, has been considered as the future for sustainable bioproduction of various target chemicals. Although historically synthetic biology approaches relied on introducing existing pathways in a specific host microorganism, recent engineering progress, as well as the development of state-of-the-art computational tools and methods have allowed the design and implementation of novel synthetic pathways, dramatically expanding the catalogue of products synthesized by chassis organisms. For example, the industrial platform chemical EG has previously been reported to be produced by *E. coli*, *S. cerevisiae,* and *C. glutamicum*; thus, progressing toward its sustainable production. The study reported here explored the possibility of producing this important target chemical in a non-model organism such as the acetogen, *C. autoethanogenum*, which provides a great opportunity for simultaneous CO_2_ fixation and EG production due to the organism’s gas fermentation capability. The described steps, illustrated in Figure 1, were followed to address a proof-of-concept approach to investigate whether an integrated workflow could be developed and implemented in *C. autoethanogenum* for the design, analysis, and expression of a *de novo* pathway for EG bioproduction in this host organism.

### Pathway Design

**Figure 2:**
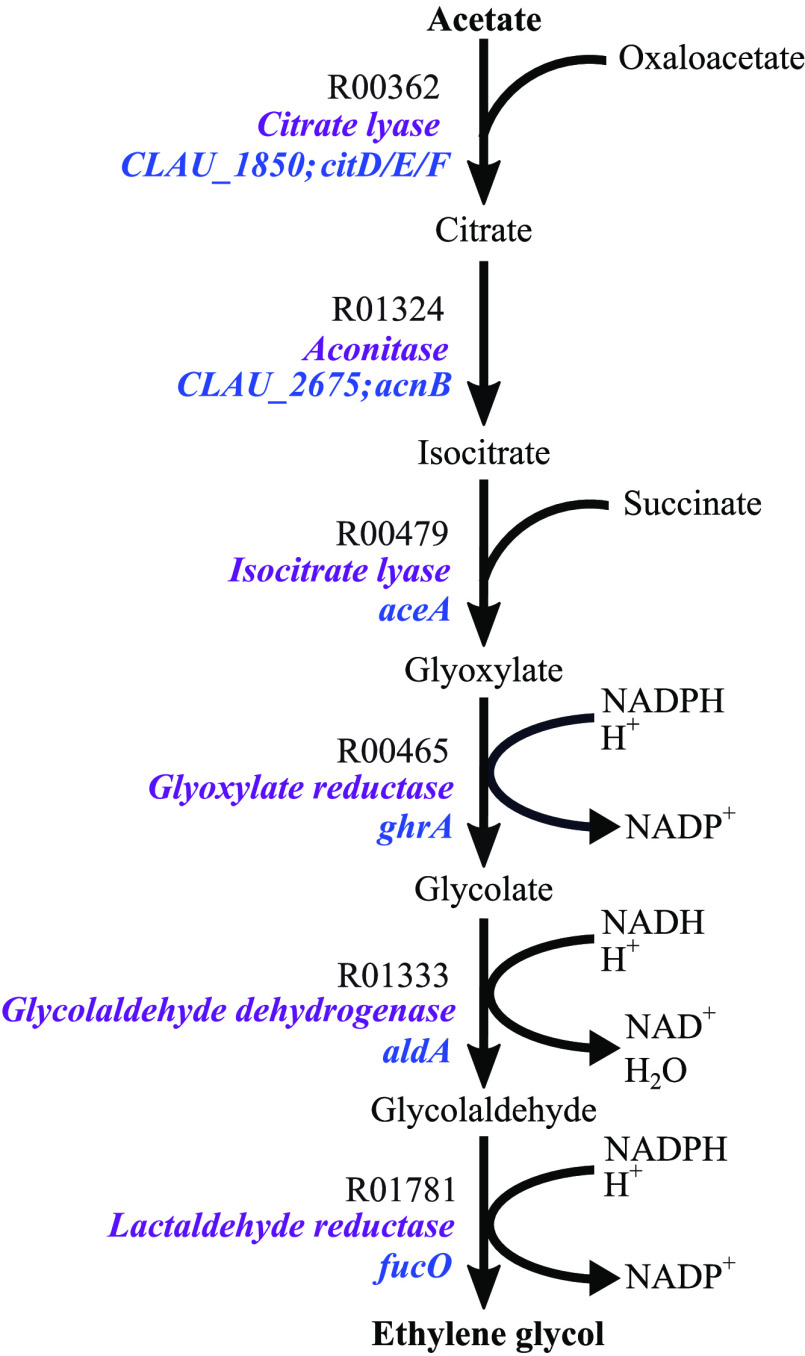
The designed biosynthetic pathway converts acetate into EG in six steps. The KEGG reaction number (R0XXXX) is listed for each step when available. The enzymes (in purple and italics) and the genes (in blue and italics) required for the steps are also shown for each step. The genes CLAU_1850 and CLAU_2675 are from *C. autoethanogenum*, while the genes *citD*/*E*/*F*, *acnB*, *aceA*, *ghrA*, *aldA*, and *fucO* are from *E. coli*.

Although myriad computational cheminformatics tools are now available to design synthetic biochemical pathways, the two tools—FMM^38^ and MRE^39^—were predominantly employed here due to their user-friendly interface with superior pathway prediction capabilities. These tools create pathways in a retrosynthetic manner,^48−50^ linking a target product, that is, EG, to a starting metabolite, that is, acetate. Acetate was chosen as the starting point due to its high production rates during gas fermentation by acetogens. The conversion of acetate into other target products would also unlikely be detrimental to *C. autoethanogenum* growth because acetate production through the conversion of acetyl-CoA via the WL pathway generates one mole of ATP under autotrophic conditions.^16,51^ In addition, both tools create pathways using only biologically known reactions listed in the Kyoto Encyclopedia of Genes and Genomes (KEGG) database.^52^ Other tools such as ATLAS of Biochemistry^53^ design pathways with novel reactions, but were not explored in this study to avoid the extensive protein engineering efforts required for experimental implementation of novel reactions. To further select the best candidates among the extensive number of pathways generated by FMM and MRE, four main pruning criteria (pathway length, requirement of external metabolites, gene availability, and gene origin) were first applied in a more rational preselection to exclude inadequate pathways (Figure 1). As such, when possible, the shortest pathways were selected to reduce suboptimal kinetics, caused by the various origins of the pathway enzymes. Similarly, pathways relying on metabolites not naturally produced by *C. autoethanogenum* metabolism were excluded to avoid medium supplementation or additional metabolic engineering efforts. Finally and arguably, the main limitation of synthetic pathway design remains gene availability of the reactions included in the pathway. Although only existing reactions from KEGG were used in the designed pathways, the genes encoding enzymes for many of these reactions have not been identified yet, preventing their insertion into a host organism. In addition, gene origin and host’s compatibility must be taken into account when inserting heterologous genes to reduce protein misfolding and inactivity, which can, for example, be mediated by codon optimization or harmonization,^21^ or site-specific protein engineering to ensure correct protein folding. In addition, the gene origin also dictates the intracellular parameters, such as pH and growth conditions (e.g., anaerobic vs aerobic), required for correct protein folding and may complicate protein expression in a phylogenetically distant organism with different intracellular conditions. As such, it is advisable to select genes from closely related organisms to the chosen host, when possible, to avoid additional protein engineering efforts, which inevitably further limits the number of candidate genes. Using these different selection criteria, several pathways (example pathways shown in the Supporting Information) were designed to convert acetate into EG in *C. autoethanogenum*. However, only the selected pathway that was further analyzed and successfully constructed in this acetogen is shown in Figure 2. This pathway was thought to be particularly promising as it satisfied all described pruning criteria and was further investigated for host compatibility as detailed below.

### Pathway Analysis

**Figure 3:**
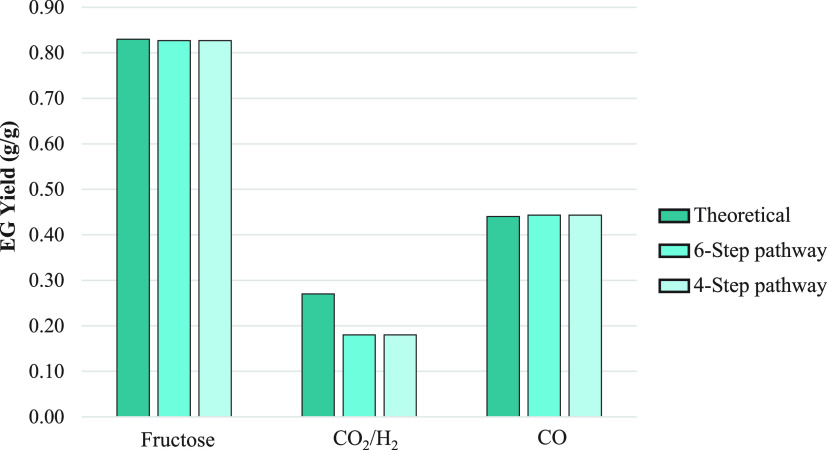
Comparison of predicted and theoretical EG yields in*C. autoethanogenum*. Predicted EG pathway yields from fructose, CO_2_/H_2_, and CO using the*C. autoethanogenum* GEM and FBA were compared with the estimated theoretical EG yields from the same substrates for the 4-step and 6-step versions of the pathway.

To predict the feasibility and yield of the selected pathway (Figure 2), it was analyzed by integrating in the genome-scale model (GEM) of*C. autoethanogenum*.^40^ Using the COBRA toolbox,^41^ FBA was performed to predict pathway yield from three substrates (fructose, CO_2_/H_2_, and CO) and compared to the theoretical yields estimated from the degree of reductance of substrates and the product, as previously described^54^ (Figure 3). Interestingly, computational analysis of heterologous expression of the first two genes, already natively present in*C. autoethanogenum*, did not lead to increased EG yield. In addition, CO was predicted to be a superior substrate than CO_2_/H_2_ for EG production during the autotrophic growth of*C. autoethanogenum*, as seen from the theoretical yields and the FBA predictions (Figure 3); this result is in accordance with previous reports.^40^ A minor overestimation of EG yield (0.4432 g EG/g CO) from CO using the*C. autoethanogenum* GEM was observed as compared to the theoretical EG yield from CO (0.44 g EG/g CO); however, this issue was not further addressed as it seemed negligible and did not influence later analyses.

**Figure 4:**
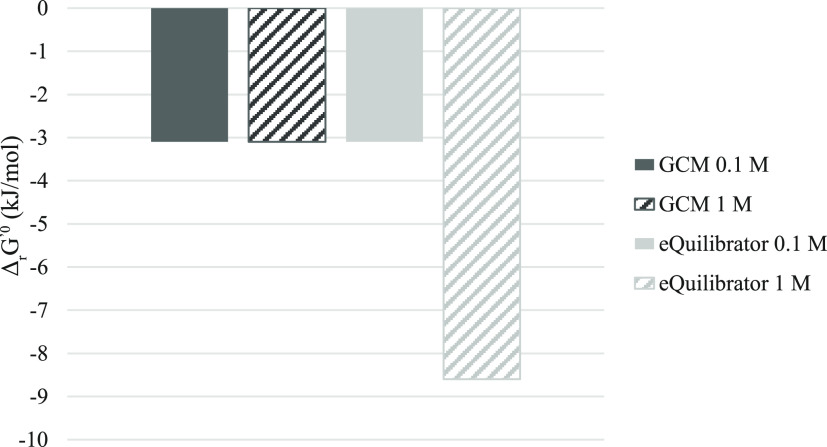
Overall standard Gibbs free energy change (Δ_r_*G*′°) values for the EG pathway, estimated with the GCM and eQuilibrator, for metabolite concentrations of 1 and 0.1 M. Notably, the method used impacts the estimated value itself although all analyses agreed that the pathway is overall thermodynamically feasible.

**Figure 5:**
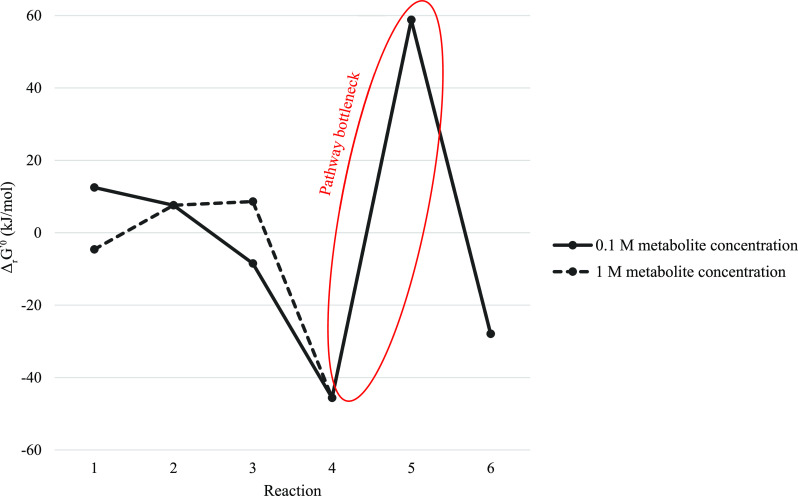
Standard Gibbs free energy change (Δ_r_*G*′°) values estimated by the group contribution method for each reaction of the pathway under standard 1 M concentrations and physiological 0.1 M concentrations of metabolites. These values indicate that reaction 5 is the main bottleneck of the pathway in terms of its thermodynamic feasibility.

To further investigate the pathway feasibility, a thermodynamics-based approach was applied. The GCM^43^ and the online tool, eQuilibrator^44^ were used to calculate the pathway’s overall thermodynamic feasibility. Although both methods agreed that the pathway had an overall negative standard Gibbs free energy change (Δ_r_*G*′°) value, the values themselves were very different depending on the metabolite concentrations or the calculation methods used (Figure 4). While calculations were performed with 0.1 and 1 M metabolite concentrations, 0.1 M was reported^55^ to represent more closely to physiological conditions, as most metabolite concentrations range between 1 and 100 mM in living cells. In addition, it has previously been reported^55^ that metabolite concentration impacts calculated Δ_r_*G*′° values as observed with these results. While both conditions are considered here to highlight that the pathway is thermodynamically feasible in both contexts, calculations with physiological conditions are more accurate for biological systems. Moreover, this type of analysis can also guide further engineering efforts. Indeed, the thermodynamic data of the reactions showed that reaction 5, converting glycolate to glycolaldehyde, is in fact a potential major bottleneck for the pathway (Figure 5) due to its positive Δ_r_*G*′° value. This observation suggests that this step would require further metabolic engineering efforts in the future, for example, by manipulating metabolic fluxes or applying protein engineering strategies, to increase EG yield, especially relevant in an industrial context. It is worth noting that the thermodynamic analyses described here were performed exclusively on the thermodynamic limitations of the pathway without considering the host’s metabolic network. In other words, investigation of the thermodynamic limitations of the pathway using the*C. autoethanogenum*GEM was not explored in detail here, but may be important for a more constrained analysis in the host’s context.

While the computational analysis described in this study remains extremely useful to test the feasibility of a pathway in the context of a specific host to allow the identification of pathway bottlenecks or other obstacles before its time-consuming implementation, there are still some aspects that cannot be predicted accurately using the *in silico* analyses. First, a clear gap between computational models and experimental implementation remains, especially for non-model organisms. For example, omics data is still missing from the GEM, which biases pathway feasibility analyses and can overlook detrimental impacts on the metabolism. In fact, pathway competition with the core metabolism might be misinterpreted. Regulation of metabolic pathways is also often overlooked in computational analysis due to the lack of adequate experimental data to support integrating these values within GEMs but has a direct and significant impact on metabolic outcomes, including target production. In addition, synthetic pathways usually rely on enzymes originating from various species, which often leads to suboptimal enzyme kinetics, greatly impacting pathway productivity. Unfortunately, this aspect is not taken into account with the computational methods described here. Similarly, enzyme interactions, possible toxicity, or inadequate gene expression levels (later suspected for *fucO* during pathway implementation) stand out as major obstacles when starting pathway implementation but are often missed during computational analysis. As such, computational analyses can undeniably act as a first compatibility assay to predict pathway feasibility or yield, but biases and gaps remain in computational methods, preventing prediction of some notable difficulties important for pathway implementation in a host organism.

### Pathway Implementation in *C. autoethanogenum*

The computational analyses described above led to the conclusion that the designed synthetic pathway for EG production would be feasible in*C. autoethanogenum*. Therefore, heterologous expression of the pathway’s genes in this host was explored and implemented. First, the genes required for expressing all the pathway enzymes were placed as a synthetic operon, under the control of the P_*tdcB*_ promoter.^46^ This lactose-inducible system allows fine-tuned target expression as previously described.^46^ For this step, the 4-step (reactions 3–6) and 6-step (reactions 1–6) versions of the pathway (Figure 2) were compared to confirm that overexpression of the first two reactions already present in *C. autoethanogenum* would not improve yield as per the computational predictions. Unfortunately, these synthetic operons did not allow EG production (data not shown). In fact, while attempting to quantify protein expression to confirm that the chosen expression system was not preventing target gene expression to synthesize EG with these operons, the *fucO* gene (Figure 2) was systematically excised from the construct when each gene of the operon carried a FLAG-tag (data not shown); therefore, leading to the conclusion that expression of all the target genes as a synthetic operon was not feasible in*C. autoethanogenum*. It was hypothesized that the *fucO* expression level induced by the initial P_*tcdB*_ promoter and the high-copy plasmid was not viable in the chosen host organism. Although expression levels seem the most likely explanation for these preliminary results, additional metabolic impacts or possible detrimental enzymatic interactions cannot be excluded to explain the results gathered with the initial synthetic operon. To overcome this obstacle, the genes, *aceA*, *ghrA*, and *aldA* from*E. coli*, corresponding to reactions 3, 4, and 5, respectively (Figure 2), were expressed as a synthetic operon controlled by the inducible P_*tcdB*_ promoter, while *fucO*, coding for the last reaction of the pathway (Figure 2), was placed under the control of the theophylline-inducible riboswitch^47^ on a separate plasmid (Figure S1 in the Supporting Information). In this approach, expression at the P_*tcdB*_ promoter was controlled by the*Clostridiodes difficile* sigma R factor TcdR, inserted in *C. autoethanogenum* genome and controlled by the lactose-inducible promoter P_*bgaL*_, itself activated by the transcriptional regulator BgaR to allow a two-level expression control, as described by Woods et al.^46^ Therefore, induction of genes regulated by P_*tcdB*_ was achieved by addition of lactose in the medium. Moreover, the riboswitch used for *fucO* expression prevented gene expression in the absence of theophylline by forming a stem loop structure sequestering the ribosome-binding sequence (RBS); thereby, preventing ribosome binding and gene expression. When theophylline was added, the RBS was released from the stem loop structure to allow for ribosome binding and gene expression. This theophylline-inducible riboswitch was fused to the strong constitutive P_*fdx*_ promoter, derived from*C. sporogenes*ferredoxin gene, as previously described by Cañadas et al.^47^ In addition, the two plasmids used for operon and *fucO* expression carried different Gram-positive replicons and selection markers to limit unwanted recombination between these two vectors (Figure S1 in the Supporting Information).

**Figure 6:**
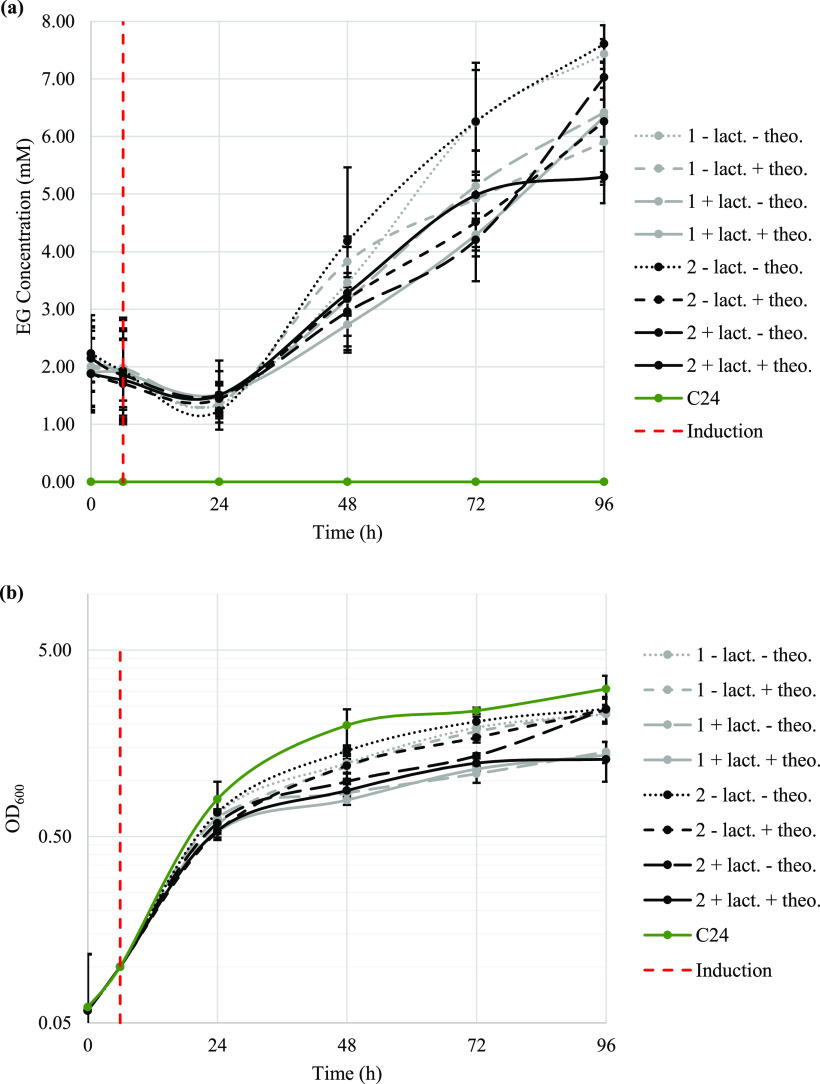
EG production profile and corresponding culture growth curves of transformants 1 and 2, with or without the presence of inducer(s) in a fructose-rich medium, are shown in (a, b), respectively. C24 represents the*C. autoethanogenum* C24 control strain carrying the regulatory elements for induction at P_*tcdB*_. Addition of the inducer(s) is represented by the red dotted line. Lact. = lactose; theo. = theophylline. Error bars represent standard deviation (*n* = 3).

**Figure 7:**
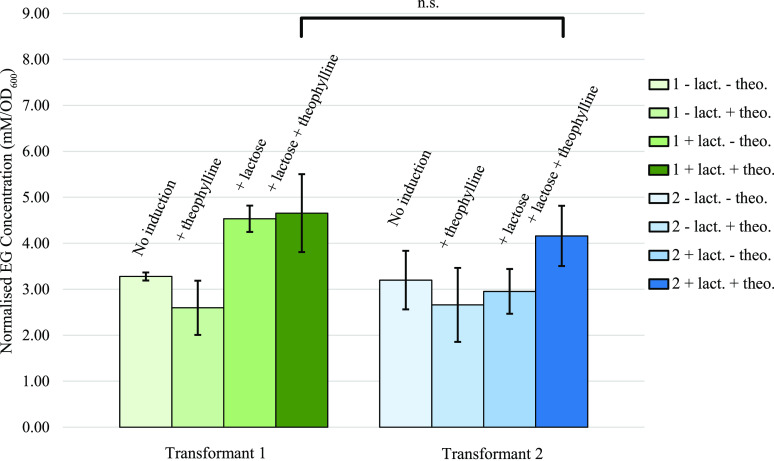
Final EG concentration at *t* = 96 h and normalized to OD_600_ of corresponding cultures. For both transformants, the highest EG concentration was reached when the two inducers (+ lactose + theophylline) were added. Lact. = lactose; Theo. = theophylline. Statistical significance was calculated with a two-tailed *t*-test. n.s. = *P* > 0.05.

This two-plasmid approach allowed EG production in*C. autoethanogenum* (Figure 6), suggesting that the original operon with all the genes led to potential negative interactions between the enzymes, or unfeasible expression levels of *fucO*. Upon conjugation of both plasmids, two transformants seemed promising as preliminary experiments showed low EG concentrations (data not shown). Simultaneous maintenance of both plasmids led to the production of EG in*C. autoethanogenum*, even when no inducer was added (Figure 6a), indicating that both promoters used allowed some levels of gene expression in the non-induced state. In fact, both promoters have previously been reported to allow low expression levels in the non-induced state,^46,47^ further suggesting that the two genetic systems used here allowed detectable EG production even without the use of inducers. As expected, the presence of the plasmids slowed down the cell growth (Figure 6b), as plasmid maintenance can be burdensome to cells. This detrimental effect was further exacerbated with the addition of inducer(s), indicating a potential toxic effect of overexpressing one or more of these genes in *C. autoethanogenum*. Notably, a significant EG concentration was detected at early timepoints although active EG production started 24 h postinoculation (Figure 6a). Indeed, some EG was carried over from the precultures, hence the starting EG concentration was observed. In addition, the EG concentration decreased in the first 24 h, which was likely due to the reversibility of the pathway reactions. It can be hypothesized that at early growth phases, the concentration of pathway intermediates was limiting; thus, forcing the pathway to convert EG into the substrate to replenish intermediate pools. Once intermediate pools are replenished during the exponential growth phase, the pathway reactions occur in the direction of EG production, leading to EG synthesis. Moreover, the EG concentrations normalized to OD_600_ (Figure 7) provided a more accurate representation of EG yields in each culture, as it takes into account the cell density, and any growth defect caused by plasmid maintenance, pathway expression, and EG production. As such, although non-induced cultures achieved a higher EG concentration (Figure 6a), these cultures grew faster and reached a higher OD_600_ than the induced cultures (Figure 6b). However, when normalized to OD_600_, that is, comparing the EG concentrations for the same number of cells, induced cultures produced the highest EG concentration (Figure 7), further highlighting that all genes must be expressed for the maximal EG production. According to these results, EG yield was 0.029 g/g fructose and 0.025 g/g fructose for transformants 1 and 2, respectively (Table 2).

**Table 2:** EG Production at *t* = 96 h for the Two Transformants with Different Inducers^a^

transformants	conditions	OD_600_	measured EG concentration (mM)	EG concentration normalized to OD (mM/OD_600_)	product (EG) yield (g/g)	% FBA
transformant 1	–lact. −theo.	2.27	7.43	3.28	0.020	2.46
	–lact. +theo.	2.30	5.90	2.57	0.016	1.93
	+lact. −theo.	1.42	6.42	4.51	0.028	3.39
	+lact. +theo.	1.37	6.34	4.64	0.029	3.49
transformant 2	–lact. −theo.	2.42	7.61	3.15	0.020	2.36
	–lact. +theo.	2.40	6.26	2.60	0.016	1.96
	+ lact. −theo.	2.41	7.03	2.91	0.018	2.19
	+lact. +theo.	1.30	5.30	4.08	0.025	3.06

a Product yield is also listed and represented as the percentage of the model-based FBA prediction. Lact = lactose; Theo = theophylline.

Although this study demonstrates that computationally designed synthetic pathways can be implemented in microorganisms, the product yields achieved represented only 3.49% (transformant 1) and 3.06% (transformant 2) of the maximum yield predicted by FBA with fructose as the main substrate (Table 2). Although transformant 1 performed slightly better than transformant 2, the difference in EG production was not statistically significant (Figure 7). Obviously, the yields reported here are significantly low and impractical in an industrial context. Thus, much more strain engineering efforts are needed to improve the novel pathway yield and the designed strain by implementing specific metabolic engineering and potential protein engineering strategies. Nonetheless, this study proves that a computationally designed synthetic pathway can be successfully implemented in a non-model organism with unique metabolic limitations. Importantly, EG production under autotrophic conditions was not discussed here, but must be achieved to fully benefit from the metabolic abilities of*C. autoethanogenum*, allowing CO_2_-fixation while sustainably producing EG. It can be anticipated that achieving target production from C1-gases might be challenging due to the highly constrained metabolism and rigid energy limitations during autotrophy. However, as mentioned previously, this study merely acted as a proof-of-concept approach to establish a systematic flow from computational pathway design to experimental strain development. As such, additional strain engineering approaches were not explored but are necessary to build a robust strain for industrial applications. For example, genome integration of the target genes was not attempted in this study but is required to avoid the need for selective pressure and increase strain stability, especially if larger-scale fermentations are considered. It would also be useful to investigate, for example, how genome integration would impact pathway yield. In addition, further strain engineering will be required to reach productivities high enough to render the strain cost-effective. This might be mediated by additional genetic engineering efforts, such as implementing different expression systems, or protein engineering to improve enzyme kinetics and alleviate potential unfavorable enzyme interactions. Finally, other metabolic engineering strategies might be beneficial, especially to bypass the identified pathway bottleneck (Figure 5) and to manipulate metabolic fluxes. Thus, the work described here serves as an example for the development of novel strains through preliminary computational analyses but does require further work to improve the engineered*C. autoethanogenum* strain for EG production.

## Conclusions

EG is an important industrial platform chemical for the production of various value-added target commodities. Due to its high demand and numerous applications, its sustainable bioproduction is crucial to reduce the demands on fossil fuels for its availability. In fact, such production has been reported for EG in three model microorganisms using several engineered metabolic pathways. Here, we described the design and implementation of a novel synthetic pathway, allowing the conversion of acetate to EG in the industrially important acetogen,*C. autoethanogenum*. Importantly, this study describes a workflow to design, prune, and analyze synthetic pathways with computational tools for a specific value-added chemical and successful implementation of the pathway expression in a microbial chassis, leading to bioproduction of the target chemical. Although the workflow described here is promising and opens the door for the bioproduction of other target chemicals in*C. autoethanogenum*, much more work is still needed to optimize the designed pathway and the engineered strain to improve yield and productivity in order to render it viable for industrial applications. Improving expression levels of different target genes seems particularly important for successful EG production as shown by the suspected unviable expression of original *fucO*. As such, other expression systems could be explored to increase gene expression levels while maintaining viability. In addition, other candidate genes from other organisms homologous to the ones implemented here might also increase pathway efficiency due to having different enzyme kinetics and substrate specificities. Similarly, rational or randomized protein engineering might be useful to further improve enzyme parameters at later stages of pathway optimization. Additional metabolic engineering strategies could also be explored, for example, to increase cofactor pools or downregulate competing pathways to achieve maximal EG production in*C. autoethanogenum*. The results described here clearly highlight the gap remaining between computational predictions and experimental implementation of synthetic metabolic pathways in a non-model chassis, which promotes additional efforts for developing more reliable data-informed computational models to truly access the full potential of synthetic biology and metabolic engineering for sustainable production of various target products, especially in non-model organisms with constrained metabolism.

## Supporting Information Available

Examples of computationally designed synthetic pathways; their limitations highlighted according to the pruning criteria; plasmid maps of the vectors; and primers used (PDF)
