# Supplementary material for: Design, Analysis, and Implementation of a Novel Biochemical Pathway for Ethylene Glycol Production in Clostridium autoethanogenum
Source: ACS Synth Biol. 2022 May 11;11(5):1790–800. doi: 10.1021/acssynbio.1c00624 (PMC9127970; doi:10.1021/acssynbio.1c00624)
Supplement: Supplementary file 1 — sb1c00624_si_001.pdf [file sb1c00624_si_001.pdf]

SUPPORTING INFORMATION

Design, Analysis and Implementation of a Novel Biochemical Pathway for Ethylene Glycol Production in *Clostridium autoethanogenum*

Barbara Bourgade[1,2], Christopher M. Humphreys[2], James Millard[2], Nigel P. Minton[2] and M. Ahsanul Islam[1]*

[1]Department of Chemical Engineering, Loughborough University, Loughborough, LE11 3TU, UK
[2]BBSRC/EPSRC Synthetic Biology Research Centre, Biodiscovery Institute, University of Nottingham, Nottingham, NG7 2RD, UK

***Corresponding author: M.Islam@lboro.ac.uk**

Figure S1. Examples of computationally designed pathways to convert acetate into EG. The blue arrows show the reactions described here for construction and implementation in *C. autoethanogenum*. Red arrow: reaction requiring acetyl-CoA; green arrow: reaction requiring an external metabolite, i.e., not natively produced by *C. autoethanogenum*; orange arrow: reaction requiring oxygen as a cofactor. The chosen pathway stands out as the best candidate pathway given its length and the cofactors involved in the reactions.

Figure S2. Plasmid 1 carried the three genes *aceA*, *ghrA* and *aldA* controlled by the P_{tcdB} promoter and was replicated in *C. autoethanogenum* with the pCB102 replicon under clarithromycin selection (encoded by the *ermB* gene). The second plasmid only harboured the *fucO* gene, controlled by the P_{fdx}-riboswitch and selected for in *C. autoethanogenum* with thiamphenicol (*catP* gene). Plasmid replication was achieved with the pCD6 replicon. Both plasmids carried the Gram-negative colE1 replicon and *traJ*, for replication in *E. coli* and plasmid transfer during conjugation, respectively. The two plasmids were maintained simultaneously in *C. autoethanogenum*.

Table S1. Primers used in this study.

Name	5'-to-3' sequence
aceA_Fwd	ATATATgagctcACTATGGAGCATCTGCAC
aceA_Rev	ATATATactagtTTAGAACTGCGATTCTTCAG
ghrA_Fwd	ATATATactagtTTTTCGGGAGTCAGTATGGATATCATCTTTTATC
ghrA_Rev	ATATATgttaacTTAGTAGCCGCGTGCGCG
aldA_Fwd	ATATATgttaacTCACAGGAGTCGCCCATG
aldA_Rev	ATATATtctagaTTAAGACTGTAAATAAACCACCTGGG
fucO_Fwd	GGAGGTAACAACAAGGCAACAAGGAGAAGGATG
fucO_Rev	ATATATgctagcTTACCAGGCGGTATGGTAAAG
P_{fdx}_Fwd	ATATATgcggccgcGTGTAGTAGCCTGTGAAATAA
P_{fdx}_Rev	CCTTCTCCTTGTTGCCTTGTTGTTACCTCCTTAGCA

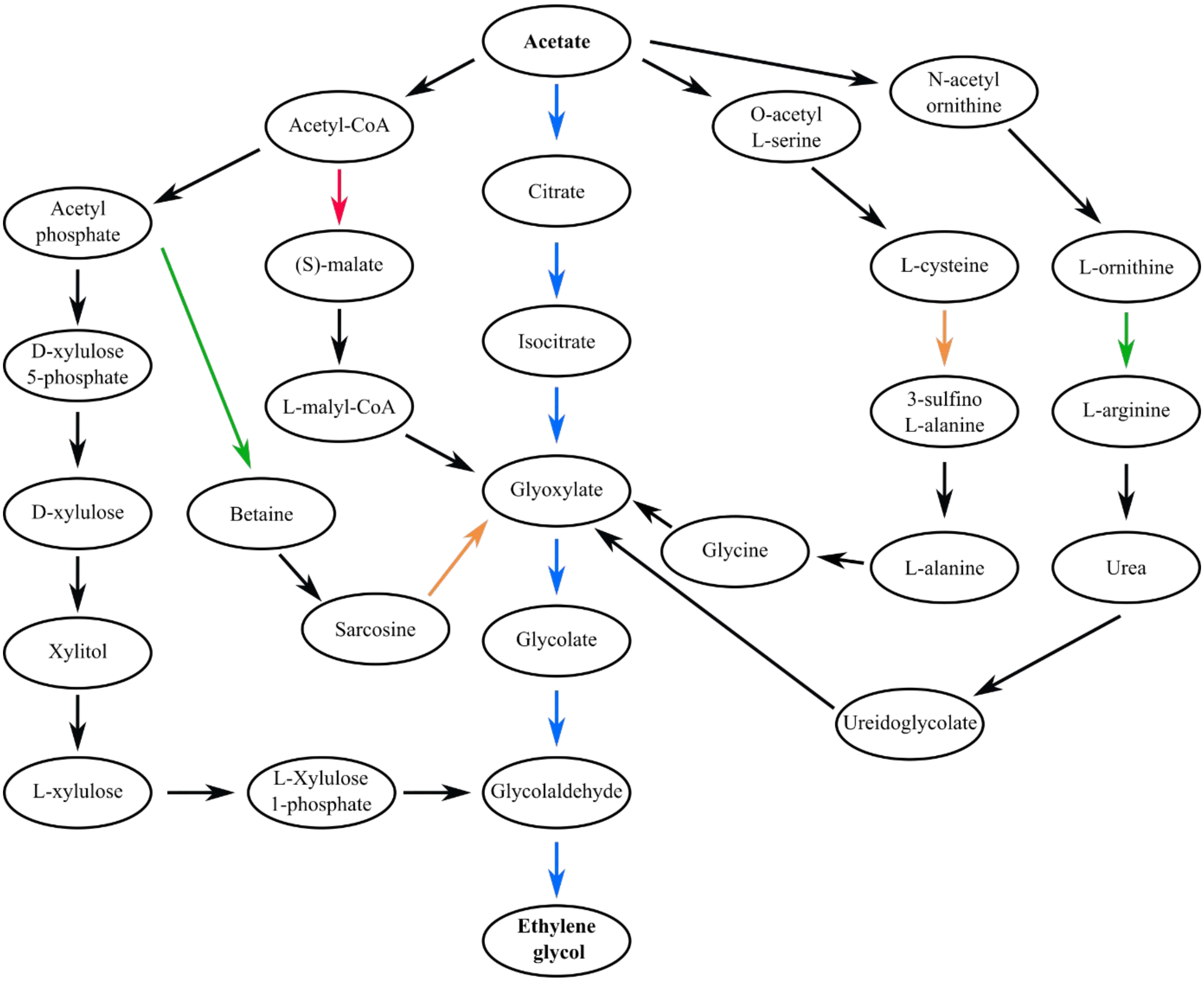
Acetate
Acetyl-CoA
Citrate
O-acetyl L-serine
N-acetyl ornithine
Acetyl phosphate
(S)-malate
Isocitrate
L-cysteine
L-ornithine
D-xylulose 5-phosphate
L-malyl-CoA
3-sulfino L-alanine
L-arginine
D-xylulose
Betaine
Glyoxylate
Glycine
L-alanine
Urea
Sarcosine
Glycolate
Xylitol
Ureidoglycolate
L-xylulose
L-Xylulose 1-phosphate
Glycolaldehyde
Ethylene glycol

Figure S1

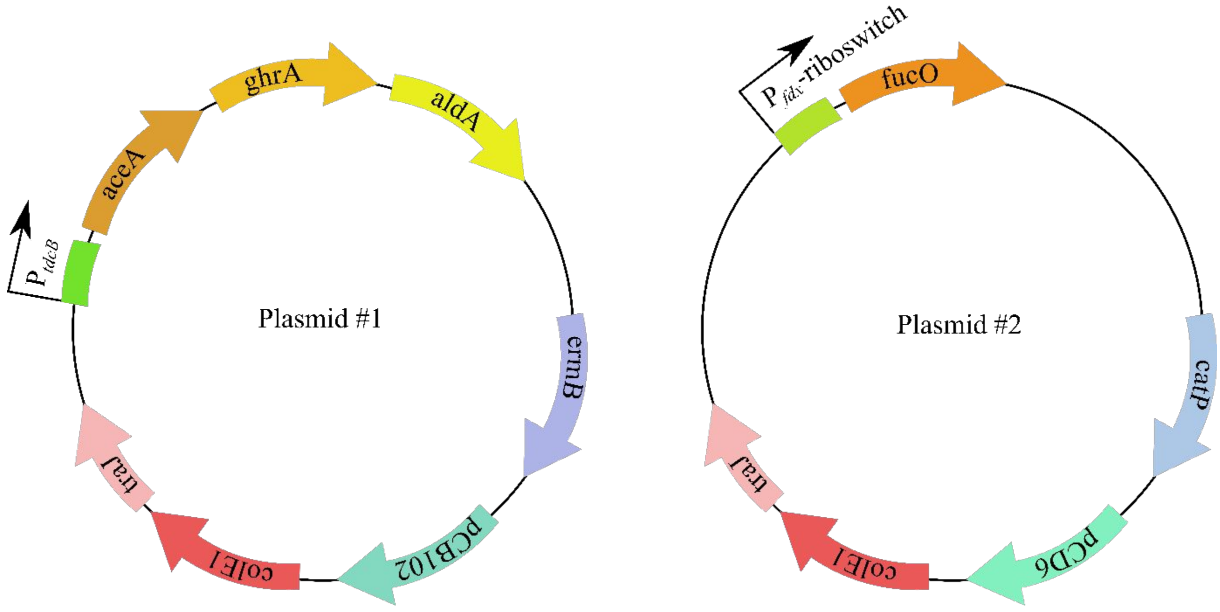

P tdcB
aceA
ghrA
aldA
ermB
pCB102
colE1
traJ
Plasmid #1
P fdx-riboswitch
fucO
catP
pCD6
colE1
traJ
Plasmid #2

Figure S2
